# Supplementary material for: Curated and harmonised transcriptomics datasets of interstitial lung diseases
Source: Data Brief. 2025 Oct 14;63:112139. doi: 10.1016/j.dib.2025.112139 (PMC12581653; doi:10.1016/j.dib.2025.112139)
Supplement: Supplementary file 1 [file mmc1.zip › Supplementary_material/RNA-seq/fastQC reports/GSE184316/TRIMMED_SRR15926764_fastqc.html]

TRIMMED\_SRR15926764.fastq.gz FastQC Report 

FastQC Report

Sat 3 Dec 2022  
TRIMMED\_SRR15926764.fastq.gz

## Summary

- Basic Statistics
- Per base sequence quality
- Per sequence quality scores
- Per base sequence content
- Per sequence GC content
- Per base N content
- Sequence Length Distribution
- Sequence Duplication Levels
- Overrepresented sequences
- Adapter Content

## Basic Statistics

| Measure | Value |
| --- | --- |
| Filename | TRIMMED\_SRR15926764.fastq.gz |
| File type | Conventional base calls |
| Encoding | Sanger / Illumina 1.9 |
| Total Sequences | 9193441 |
| Sequences flagged as poor quality | 0 |
| Sequence length | 35-373 |
| %GC | 49 |

## Per base sequence quality

## Per sequence quality scores

## Per base sequence content

## Per sequence GC content

## Per base N content

## Sequence Length Distribution

## Sequence Duplication Levels

## Overrepresented sequences

| Sequence | Count | Percentage | Possible Source |
| --- | --- | --- | --- |
| GGAAGACAGTGTGGCGATTCCTCAGGGATCTAGAACTAGAAATACCATTG | 142222 | 1.5469942103288639 | No Hit |
| GAAGGACCTCTTCAAGGAGAACTACAAACCACTGCTCAAGGAAATAAAAG | 117694 | 1.2801953044567316 | No Hit |
| ATTGATTCTTCCTACCCATGAGCATGGAATGTTCTTCCATTTGTTTGTAT | 100600 | 1.0942583957410505 | No Hit |
| ATAGCAGCATGATTTATAGTCCTTTGGGTATATACCCAGTAATGGGATGG | 89600 | 0.9746078753319894 | No Hit |
| GAAGGACCTCTTCAAGGAGAACTACAAACCACTGCTCAATGAAATAAAAG | 72017 | 0.7833519571181237 | No Hit |
| ATAGCAGCATGATTTATAATCCTTTGGGTATATACCCAGTAATGGGATGG | 41953 | 0.4563362075201223 | No Hit |
| CAGTGGGGCCACGAGCTGAGTGCGTCCTGTCACTCCACTCCCATGTCCCT | 29824 | 0.3244051927890765 | No Hit |
| ATTGATTCTTCCTACCCATGAGCATGGAATGTTCTTCCATTTGTTTGTGT | 26138 | 0.28431139113200377 | No Hit |
| CACCTCCCCGAAGCTCAGGGAGAGCCCTGTTAGGGCCGCCTCTGGCCCTA | 24941 | 0.27129123904749053 | No Hit |
| GGAAGACAGTGTGGCGATTCCTCAAGGATCTAGAACTAGAAATACCATTG | 23015 | 0.25034152065586757 | No Hit |
| TTCAGCCCTGATCAAGACATGAGGGAGGCAGGGGCTCAGCTGAAGAAGCT | 19714 | 0.21443548721311206 | No Hit |
| AGGCTACACACTGAGGACTCTGTTCCTCCCCTTTCCGCCTAGGGGAAAGT | 19070 | 0.2074304931091634 | No Hit |
| GGAAGACAGTGTGGTGATTCCTCAGGGATCTAGAACTAGAAATACCATTG | 18750 | 0.2039497506972634 | No Hit |
| GCATGTGTCTTTATAGCAGCATGATTTATAGTCCTTTGGGTATATACCCA | 18611 | 0.20243780321209437 | No Hit |
| GGCCATTTTCATGATATTGATTCTTCCTACCCATGAGCATGGAATGTTCT | 18385 | 0.19997952888369 | No Hit |
| ATAGCAGCATGATTTATAGTCATTTGGGTATATACCCAGTAATGGGATGG | 18224 | 0.19822828035770285 | No Hit |
| GGAAGACAGTGTGGCGATTCCTCAGGGATCTAGAACTGGAAATACCATTG | 16343 | 0.1777680413677534 | No Hit |
| GGGAAGCGGGGAATGTCTACGTGCGTATGCACGTGGCACTCTCTGCCCGA | 14928 | 0.16237663351513323 | No Hit |
| GGAAGACAGTGTGGCAATTCCTCAGGGATCTAGAACTAGAAATACCATTG | 14530 | 0.15804746014033266 | No Hit |
| ATTGATTCTTCCTATCCATGAGCATGGAATGTTCTTCCATTTGTTTGTGT | 13917 | 0.1513796629575368 | No Hit |
| GAAGGACCTCTTCAAGGAGAACTACAAACCACTGCTCAACGAAATAAAAG | 13614 | 0.148083834986269 | No Hit |
| GCCGACAAGACCAACGTCAAGGCCGCCTGGGGTAAGGTCGGCGCGCACGC | 13137 | 0.14289535332853062 | No Hit |
| TTTTCCATGAGCTTAATGATGCTTTCTCTGGGCTTTTGGGGGAGGGTGTC | 12801 | 0.13924057379603566 | No Hit |
| GGAAGACAGTGTGGTGATTCCTCAAGGATCTAGAACTAGAAATACCATTG | 12668 | 0.13779389023108973 | No Hit |
| CCCAAGTAGCTGGGACTACAGGCGCCCGCCACTACGCCCGGCTAATTTTT | 12542 | 0.13642334790640415 | No Hit |
| TGATATGGTTCCTGGCAAGCCCATGTGTGTTGAGAGCTTCTCAGACTATC | 12149 | 0.1321485611317895 | No Hit |
| AGGTCTTGGTGGTGGGGAAGGACAGGAACATCCTCTCCAGGGCCTCCGCA | 11786 | 0.1282000939582905 | No Hit |
| TGATGACACCCACCGCAACTGTCTGTCTCATATCACGAACAGCAAAGCGA | 11499 | 0.12507830310761772 | No Hit |
| GCATGTGTCTTTATAGCAGCATGATTTATAATCCTTTGGGTATATACCCA | 11012 | 0.11978104824950744 | No Hit |
| CCCTGGCTGCTACAGGAGAATAGCAGACAGGTATAGTTAAGAAACAACTT | 10957 | 0.11918279564746215 | No Hit |
| GGAAGACAGTGTGGCAATTCCTCAAGGATCTAGAACTAGAAATACCATTG | 10831 | 0.11781225332277653 | No Hit |
| AGCTGCTAAGTGCTGTGTTGTCGTTCCCCCTGCTTAAAATAAAGTTGTTT | 10708 | 0.11647434295820248 | No Hit |
| TTACAAAAGCAGAATAAAAGCGAAAAGAAATGAAAATGTTACACTACATT | 9708 | 0.10559702292101511 | No Hit |
| ATTGATTCTTCCTACCCATGAGCATGGAATGTTCTTCCATTTCTTTGTAT | 9524 | 0.10359559603417262 | No Hit |
| GGAAGACAGTGTGGCGATTCCTCAGGGATCTAGAACTAGAAATACCATTT | 9419 | 0.10245347743026793 | No Hit |

## Adapter Content

Produced by FastQC (version 0.11.7)
